# An oral WT1 protein vaccine composed of WT1-anchored, genetically engineered *Bifidobacterium longum* allows for intestinal immunity in mice with acute myeloid leukemia

**DOI:** 10.1007/s00262-022-03214-4

**Published:** 2022-06-14

**Authors:** Natsuki Nakagawa, Yoshiko Hashii, Hisako Kayama, Ryu Okumura, Hiroko Nakajima, Hikaru Minagawa, Soyoko Morimoto, Fumihiro Fujiki, Jun Nakata, Toshiro Shirakawa, Takane Katayama, Kiyoshi Takeda, Akihiro Tsuboi, Keiichi Ozono

**Affiliations:** 1 Department of Pediatrics, Osaka University Graduate School of Medicine, Suita, Osaka Japan; 2 Department Pediatrics, Osaka International Cancer Institute, Osaka, Japan; 3 Department of Microbiology and Immunology, Graduate School of Medicine, Osaka University, Suita, Osaka Japan; 4 WPI Immunology Frontier Research Center, Osaka University, Suita, Osaka Japan; 5 Institute for Advanced Co-Creation Studies, Osaka University, Suita, Osaka Japan; 6 Department of Cancer Immunology, Osaka University Graduate School of Medicine, Suita, Osaka Japan; 7 Department of Cancer Stem Cell Biology, Osaka University Graduate School of Medicine, Suita, Osaka Japan; 8 Department of Clinical Laboratory and Biomedical Sciences, Osaka University Graduate School of Medicine, Suita, Osaka Japan; 9 Division of Advanced Medical Science, Kobe University Graduate School of Science, Technology and Innovation, Kobe, Japan; 10 Division of Integrated Life Science, Graduate School of Biostudies, Kyoto University, Kyoto, Japan; 11 Department of Cancer Immunotherapy, Osaka University Graduate School of Medicine, Suita, Osaka Japan

**Keywords:** Oral anticancer vaccine, *Bifidobacterium longum*, WT1, Intestinal immunity, Antitumor activity

## Abstract

Wilms’ tumor 1 (WT1) is a promising tumor-associated antigen for cancer immunotherapy. We developed an oral protein vaccine platform composed of WT1-anchored, genetically engineered *Bifidobacterium longum* (*B. longum*) and conducted an in vivo study in mice to examine its anticancer activity. Mice were orally treated with phosphate-buffered saline, wild-type *B. longum*105-A, *B. longum* 2012 displaying only galacto-N-biose/lacto-N-biose I-binding protein (GLBP), and WT1 protein- and GLBP-expressing *B. longum* 420. Tumor size reduced significantly in the *B. longum* 420 group than in the *B. longum* 105-A and 2012 groups (*P* < 0.00 l each), indicating *B. longum* 420’s antitumor activity via WT1-specific immune responses. CD8^+^ T cells played a major role in the antitumor activity of *B. longum* 420. The proportion of CD103^+^CD11b^+^CD11c^+^ dendritic cells (DCs) increased in the Peyer’s patches (PPs) from mice in the *B. longum* 420 group, indicating the definite activation of DCs. In the PPs, the number and proportion of CD8^+^ T cells capable of producing interferon-gamma were significantly greater in the *B. longum* 420 group than in the *B. longum* 2012 group (*P* < 0.05 or < 0.01). The production of WT1-specific IgG antibody was significantly higher in the *B. longum* 420 group than in the 2012 group (*P* < 0.05). The *B. longum* 420 group showed the most intense intratumoral infiltration of CD4^+^ and CD8^+^ T cells primed by activated DCs in the PPs of mice in the *B. longum* 420 group. Our findings provide insights into a novel, intestinal bacterium-based, cancer immunotherapy through intestinal immunity.

## Introduction

*Bifidobacterium longum* (*B. longum*) strains have been shown to stimulate CD4^+^ T cells and promote Th1 responses [1]. Probiotic bacteria, when taken into dendritic cells (DCs), serve to CD8^+^ T cell priming [1–4]. These players are the constituents of CD4^+^ T cell help in cancer immunotherapy. We developed an oral vaccine platform using *B. longum* as an antigen-delivering vehicle to the intestinal immune system and generated a Wilms’ tumor 1 (WT1) oral anticancer vaccine that expresses WT1 protein—a tumor-associated antigen (TAA) [5]. WT1, which is overexpressed in most adult and pediatric patients with acute leukemia, chronic myelocyte leukemia, and myelodysplastic syndrome [6], is considered most promising among 75 TAAs as a target for cancer vaccines and/or T cell adaptive immunotherapy [7].

The plasmid carrying galacto-*N*-biose/lacto-*N*-biose I-binding protein (GLBP)-murine WT1 fusion gene” was introduced into a wild-type *B. longum* 105-A by electroporation to produce *B. longum* 420, i.e., a product of pharmaceutical devisal consisting of WT1-anchored, genetically engineered *B. longum* to augment the efficiency of priming of T cells by professional antigen-presenting cells, dendritic cells (DCs). GLBP was used to express WT1 protein on the *B. longum* surface.

The objectives of the present study were (1) to identify which immunocompetent cells in the intestinal lymphatic system (Peyer’s patches [PPs], lamina propria, and mesenteric lymph nodes [MLNs]) are activated by the vaccine and (2) to examine how the vaccine is involved in the cellular mechanisms of intestinal immunity that result in exerting its antitumor activity on a target tumor via the systemic circulation.

## Materials and methods

### Animals and study design

C57BL/6 J (H-2D^b^) mice (5–6 weeks old; female; 3–17 animals per study group) were purchased from CLEA Japan, Inc. Body weight of tumor inoculated animals was measured at specified time points. All animal studies were conducted in compliance with regulatory and academic regulations (including humane endpoints) and were approved by Osaka University Animal Experiment Committee.

### Cells

C1498, a murine leukemia cell line of C57BL/6 origin without expression of WT1 protein, was obtained from ATCC (Rockville, MD, USA). Murine WT1-expressing C1498 (C1498-mWT1) was generated by transducing C1498 cells with CMV promotor-driven murine WT1 17AA(+) KTS(+) isoform full-length cDNA that had been inserted into the pcDNA3.1(+) mammalian expression vector (Invitrogen, Tokyo, Japan).

### WT1 oral anticancer vaccines using wild-type *B. longum *105-A and genetically engineered *B. longum*

Genetically engineered *B. longum* 2012 displays only GLBP on the bacterial cell surface, while *B. longum* 420 displays a partial murine WT1 protein (117–419 amino acid residues) via GLBP. A plasmid carrying the GLBP-coding gene was introduced into wild-type *B. longum* 105-A by electroporation to generate *B. longum* 2012, while another plasmid carrying the GLBP-murine WT1 fusion gene was used to generate *B. longum* 420 [5]. *B. longum* 105-A and *B. longum* 2012 were used as controls. *B. longum* 105-A was cultured anaerobically in the Gifu Anaerobic Medium (GAM) broth (Nissui, Tokyo, Japan) at 37 °C. *B. longum* 2012 and *B. longum* 420 were cultured anaerobically in the GAM broth with 15 µg/ml spectinomycin (Sigma-Aldrich, St. Louis, MO) at 37 °C. All these three strains were washed with phosphate-buffered saline (PBS) and were then suspended to obtain the final cell density of 2 × 10^9^ colony-forming units (CFUs)/100 µl.

### In vivo leukemia cell inoculation

2 × 10^5^ C1498-mWT1 cells in 50 µl of PBS were inoculated subcutaneously to the right dorsal side of C57BL/6 J mice on day 0. Tumor growth was monitored every 2 days. Tumor volume was calculated using the following formula: (longest diameter) × (shortest diameter) × (height) × 1/2.

### In vivo CD8^+^ T cell depletion study

To deplete CD8^+^ T cells, the anti-CD8 monoclonal antibody (mAb) was prepared using the hybridoma clones in vivo in BALB/c nude mice. C57BL/6 J mice were intraperitoneally injected with 500 mg of the anti-CD8 mAb for 3 consecutive days (days -8, -7, and -6) and day -3 before day 0 when leukemia cells were subcutaneously inoculated and every 3 days since day 0 [8–10]. On day -2, CD8^+^ T cell depletion was verified by the flow cytometry (FCM) analysis of peripheral blood mononuclear cells (PBMCs) collected from the tail vein, and the analysis was conducted every 7 days since day 0 to confirm the sustained depletion of CD8^+^ T cells. For the analysis, T cells were stained with the anti-CD8-Alexa Fluor 647 (KT15, MBL, Nagoya, Japan), anti-CD3-FITC (17A2, eBioscience, San Diego, CA), anti-CD4-V500, (RPA-T4, BD Biosciences, Franklin Lakes, NJ), and anti-CD8a-V450 (53-6.7, BD Biosciences) mAbs. Tumor growth was monitored every 2 days.

### Immunohistochemical staining

Paraffin-embedded tumor tissues were used in the immunohistochemical staining of CD4^+^ and CD8^+^ T cells. A microtome (Leica, Wetzlar, DEU) was used to prepare the 4-µm-thick tissue sections before deparaffinization and rehydration. Antigen epitopes were retrieved by autoclaving at 121 °C for 20 min in 10 mM sodium citrate buffer (pH 6.0). The sections were incubated overnight at 4 °C with the rabbit anti-CD4 (Bioss, Boston, MA) or anti-CD8 (Abcam, Cambridge, UK) antibody that was diluted 1:100. Subsequently, the sections were incubated for 60 min at room temperature with the secondary antibody HRP-conjugated anti-rabbit IgG (H + L) (Promega, Madison, WI). Positive signals were visualized by using 3,3’-diaminobenzidine (Sigma-Aldrich) for 3 min before the sections were counterstained with hematoxylin for 5 min. As negative controls, the sections were stained in parallel with isotype control, the rabbit monoclonal IgG antibody (Abcam). CD4- or CD8-positive cells were counted as described previously [11].

### WT1 tetramer assay

PBMCs were collected from the tail vein of 5 mice in each study group to determine the frequency of CD8^+^ T cells recognizing the WT1 epitope WT1_126_ (a.a.126-134). The multicolor staining of PBMCs was conducted with a PE-conjugated WT1_126-134_ tetramer (MBL). Cells were incubated with anti-CD8-Alexa Fluor 647 (MBL) and anti-CD3-FITC (eBioscience), and then with 7-AAD (BD Biosciences). Fluorescence was detected and analyzed using the FACSCanto™ II (BD Biosciences) flow cytometer and the FlowJo™ software (BD Biosciences).

### Chromium 51 (^51^Cr) release cytotoxicity assay

C1498-mWT1 tumor-bearing mice received PBS, *B. longum* 105-A, *B. longum* 2012, or *B. longum* 420 on days 1 to 10; animals were killed on day 11 after leukemia cell inoculation. The cytotoxic activity of WT1-specific cytotoxic T cells (CTLs) was evaluated in the ^51^Cr release assay as described previously [12, 13]. An H-2D^b^-binding peptide, WT1_126-134_ peptide (a.a.126-134; Scrum, Tokyo, Japan), was dissolved in PBS; splenocytes were collected and stimulated with 5 μg/ml WT1_126-134_ peptide and were then cultured in 10% fetal bovine serum (FBS; Thermo Fischer Scientific, Waltham, MA), 45% RPMI 1640 (Sigma-Aldrich), 45% AIM-V (Gibco, Waltham, MA), 1 × nonessential amino acid (Gibco), 25 ng/ml 2-mercaptoethanol (FUJIFILM Wako Pure Chemical, Osaka, Japan), and 50 IU/ml penicillin-50 μg/ml streptomycin (FUJIFILM Wako Pure Chemical). Two and 4 days later, recombinant interleukin-2 (rIL-2; Kyowa Pharmaceutical Industry, Osaka, Japan) was added to the culture at a concentration of 20 IU/ml. After 6-day culture, the ^51^Cr release cytotoxicity assay was conducted to examine the WT1-specific cytotoxic activity of splenocytes on target RMAS cells with or without the addition of WT1_126-134_ peptide. The percentage of specific lysis (% specific lysis [count per minute of experimental release—count per minute of spontaneous release]/[count per minute of maximal release—count per minute of spontaneous release] × 100) was calculated at various effector/target (E/T) ratios for WT1_126-134_-pulsed and -unpulsed RMAS cells that were lysed by splenocytes from mice in the PBS, *B. longum* 105-A, *B. longum* 2012, and *B. longum* 420 groups.

### Enzyme-linked immune sorbent assay (ELISA) for anti-WT1 IgG antibody titers

WT1-specific IgG antibodies were assayed by ELISA. Briefly, 96-well plates were filled with WT1 protein (MyBioSource, San Diego, CA) and were then incubated at 4 °C. After incubation, the plates were washed with tris-buffered saline with 0.05% Tween 20 (TBST; Sigma) and were then blocked with gelatin-containing TBST for 2 h at room temperature. C57BL/6 J mice were treated with 100 µl of PBS, *B. longum* 105-A, *B. longum* 2012, or *B. longum* 420 (2 × 10^9^ CFUs/100 µl of PBS) every day. On day 28, mice plasma was collected and diluted to 1:1000 with TBST, and 100 µl of diluted plasma were added to each well for incubation at 4 °C. All samples were measured in duplicate wells. Absorbance at a wavelength of 550 nm was recorded using the MTP-310 microplate reader (Hitachi, Tokyo, Japan). ELISA endpoint titers were defined as “the mean absorbance in two wells for a single sample containing serum”—“the absorbance in a well for a single sample that was free of serum.” 6F-H2, an anti-WT1 antibody (Millipore, Temecula, CA), was used as positive control, and buffer was used as negative control.

### Multicolor fluorescence in situ hybridization (FISH) for the detection of *B. longum*

C57BL/6 J mice were orally given 100 µl of PBS or *B. longum* 420 (2 × 10^9^ CFUs/100 µl of PBS) every day for 10 days. On day 11, the small intestine was removed and then fixed with the Carnoy’s solution before paraffin embedding. The rRNA sequence specific to *B. longum* (Blon1004) (AGCCGTATCTCTACGACCGT) was labeled with Cy3 at the 5’ end (Sigma-Aldrich) [14]. The Cy5-labeled oligonucleotide probe Eub338—which is complementary to a region of 16S rRNA highly conserved in the domain *Bacteria* [15]—was also used to detect the presence of *B. longum* 420 in the lumen of the small intestine and the PPs. The 100-µl solution of hybridization containing 1 µg each of these probes was poured onto each section. The sections were mounted with the VECTASHIELD® Mounting Medium with DAPI (Vector Laboratories, Burlingame, CA). MojoSort™ Mouse CD11c Nanobeads (Biolegend, San Diego, CA) were used to positively sort CD11c^+^ DCs in the PPs from mice that had been orally given *B. longum* 420 for 15 days. The resulting DCs were separated into two aliquots for May-Giemsa staining and FISH.

### Intracellular cytokine staining

C57BL/6 J mice were orally given 100 µl of PBS, *B. longum* 2012, or *B. longum* 420 (2 × 10^9^ CFUs/100 µl of PBS) every day. On day 29, animals (*n* = 8/group) were killed to remove the small intestine including the PPs, MLNs, and colon. The expressions of interferon (IFN)-γ^+^CD4^+^ and IFN-γ^+^CD8^+^ T cells in the PPs, MLNs, small intestine, and colon were analyzed with the Cytofix/Cytoperm™ Plus Kit with GolgiStop™ (BD Biosciences) according to the manufacturer’s instructions. In brief, the cells were incubated with 50 ng/ml PMA (phorbol 12-myristate 13-acetate; Sigma-Aldrich), 5 μM calcium ionophore A23187 (Sigma-Aldrich), and GolgiStop™ in RPMI 1640. Surface staining was conducted with the anti-CD8-APC-Cy7 antibody (53-6.7, BD Biosciences) and the anti-CD4-Percp-Cy5.5 antibody (CK1.5, BD Biosciences), and intracellular cytokine staining was conducted with the FITC-conjugated anti-IFN-γ antibody (XMG1.2, BD Biosciences) [16]. Samples were analyzed using the FACSCanto™ II flow cytometer and the FlowJo™ software.

### Flow cytometry of the PPs, MLNs, and spleen

C57BL/6 J mice were orally given 100 µl of PBS, *B. longum* 105-A, *B. longum* 2012, or *B. longum* 420 (2 × 10^9^ CFUs/100 µl of PBS) every day for 10 days. On day 11, animals (*n* = 8/group) were killed to remove the small intestine including the PPs, MLNs, and spleen. Cells in the PPs, MLNs, and spleen were incubated with the following antibodies: anti-CD45-APC-Cy7 (30-F11, BD Biosciences), anti-CD11c-PE-Cy7(HL3, BD Biosciences), anti-major histocompatibility (MHC) class I-A/I-E (MHC II)-BV421 (M5/114.15.2, BD Biosciences), anti-CD103-APC (M290, BD Biosciences), anti-CD11b-BV510 (M1/70, BD Biosciences), and 7-AAD (BD Biosciences), and anti-CD86-FITC (GL-1, Biolegend). Samples were analyzed using the FACSCanto™ II flow cytometer and the FlowJo™ software. MHC class II^+^CD11c^+^ cells were gated from 7-AAD^−^CD45^+^ cells, followed by the analysis of DC activation based on the measured levels of CD86 and by the subset analysis of DCs through the levels of CD103 and CD11b [17, 18].

### Statistical analysis

Comparisons among multiple groups were conducted by one-way analysis of variance, followed by Student’s t test. A *P* value of < 0.05 was considered statistically significant. All statistical analyses were conducted with JMP® version 14.1 (SAS Institute Inc., Cary, NC) and the SAS software version 9.4 (SAS Institute Inc.).

## Results

### In vivo antitumor activity of *B. longum*

**Fig. 1:**
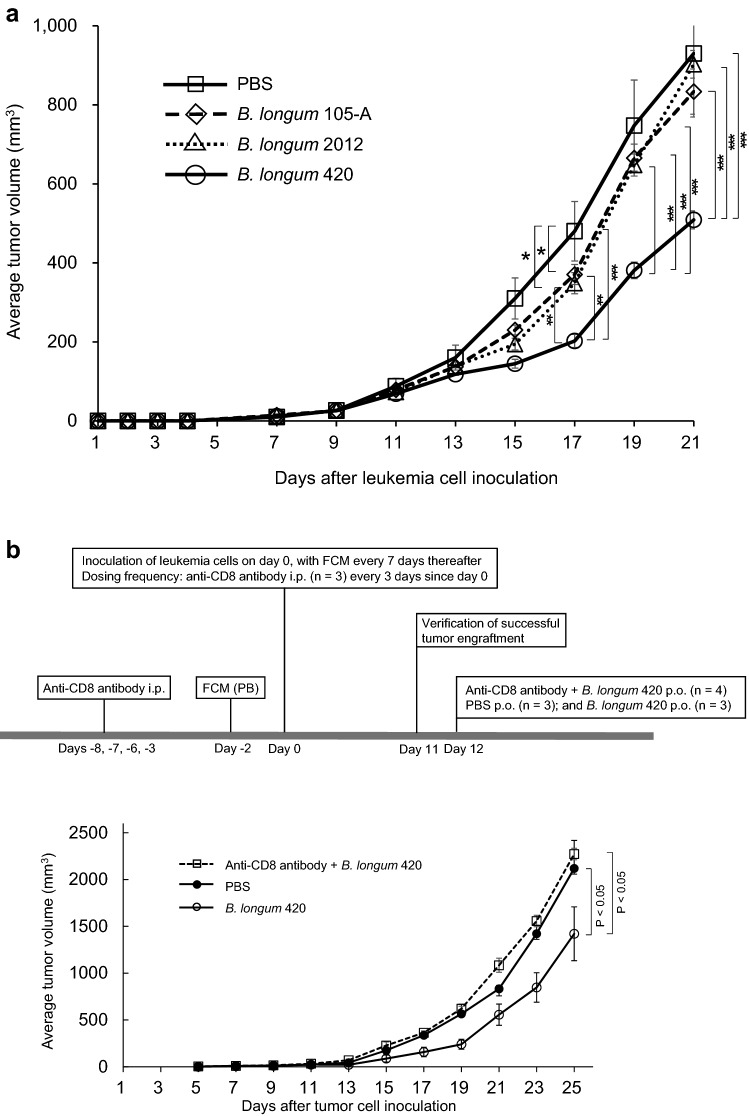

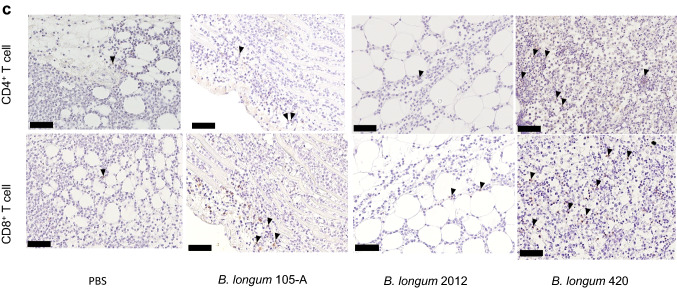

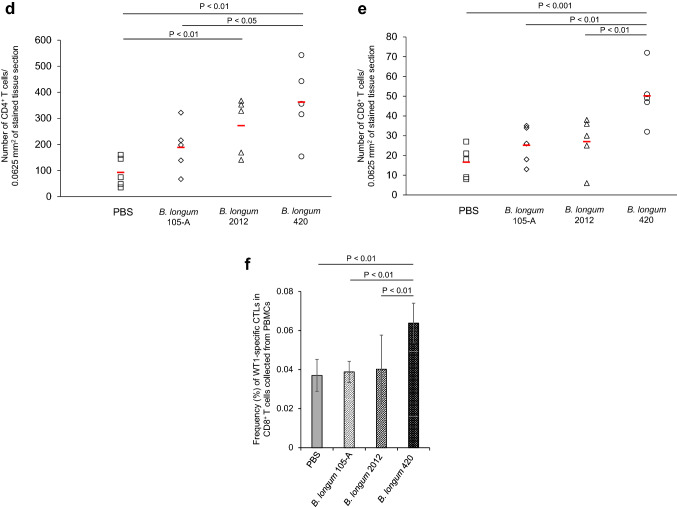

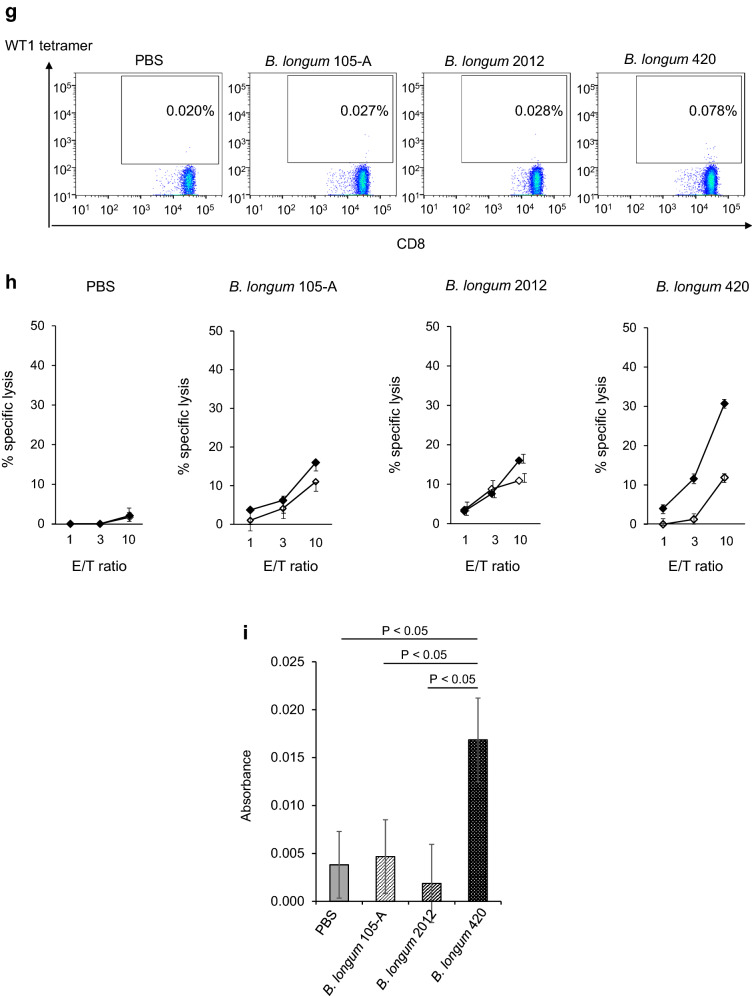
Antitumor activity of study materials, intratumoral infiltration and number of T cells, % specific lysis of target RMAS cells by splenocytes, frequency of WT1-specific CTLs, and anti-WT1 IgG antibody titers. **a** Average tumor volume changes after leukemia cell inoculation (*n* = 17 each). Scale bars represent standard error. *: *P* < 0.05, **: *P* < 0.01, ***: *P* < 0.001. Two-sided Student’s t test, except for day 21 that was analyzed by Dunnett test. Successful tumor engraftment was verified on day 10 when the oral administration of study materials was started. **b** The upper diagram showing the study schedule in mice, including the frequency of FCM. The lower diagram showing average tumor volume changes after leukemia cell inoculation with or without the depletion of CD8^+^ T cells, followed by the oral administration of PBS and *B. longum* 420 starting on day 12. Scale bars represent standard error. Two-sided Student’s t test. **c** After tumor inoculation on day 0, oral administration of study materials was conducted on days 1 to 28. Animals were killed to remove the tumor. Immunohistochemical stains of TILs. Images are representative of 2 and 3 experiments for CD4^+^ and CD8^+^ T cells, respectively. Scale bar, 50 µm. Arrowheads, tumor-infiltrating CD4^+^ and CD8^+^ T cells. **d** Number of CD4^+^ T cells in the study groups (*n* = 5 each). Red lines denote the mean values. **e** Number of CD8^+^ T cells in the study groups (*n* = 5 each). Red lines denote the mean values. **f** Successful tumor engraftment was verified on day 10 when the oral administration of study materials was started. On day 18 after leukemia cell inoculation into animals of the study group (*n* = 5 each), the tetramer assay was conducted to determine the frequency (%) of WT1-specific CTLs whose T cell receptor recognize a complex consisting of H-2D^b^ and the WT1 epitope WT1_126_ (a.a.126-134)—RMFPNAPYL. **g** Representative diagrams of flow cytometry plotting WT1 tetramer-positive CD8^+^ T cells in mice treated with PBS, *B. longum* 105-A, *B. longum* 2012, and *B. longum* 420. **h** C1498-mWT1 cells were subcutaneously inoculated on day 0. Oral administration was conducted on days 1 to 10. On day 11, % specific lysis of WT1_126-134_ peptide-pulsed target RMAS cells (a TAP-deficient subline of RMA—Rauscher leukemia virus-induced lymphoma cell line of C57BL/6 origin) by splenocytes was determined. Solid diamond (WT1_126-134_ peptide-pulsed RMAS cells), open diamond (WT1_126-134_ peptide-unpulsed RMAS cells). Mice treated with PBS, *B. longum* 105-A, *B. longum* 2012, and *B. longum* 420. Representative data in triplicate are shown. **i** Oral administration was conducted on days 1 to 28. The anti-WT1 IgG antibody titer, expresses as absorbance at a wavelength of 550 nm, was significantly greater in the *B. longum* 420 (*n* = 7) group than in the PBS (*n* = 11), *B. longum* 105-A (*n* = 9), and *B. longum* 2012 (*n* = 8). PBS, phosphate-buffered saline; *B. longum*, *Bifidobacterium longum*; FCM, flow cytometry; i.p., intraperitoneal; PB, peripheral blood; p.o. *per os*; CTLs, cytotoxic T cells; PBMCs, peripheral blood mononuclear cells; E/T, effector target ratio

We examined the anticancer activity of the following materials: controls that did not express WT1 protein—PBS, wild-type *B. longum* 105-A, and *B. longum* 2012 displaying only GLBP; and WT1 protein- and GLBP-anchored *B. longum* 420 on the bacterial cell surface. Subsequent to day 10 when successful tumor engraftment was verified, mice received PBS, *B. longum* 105-A, *B. longum* 2012, and *B. longum* 420. Body weights of any animals in the study groups did not decrease during the study period. The average volume of the C1498-mWT1 tumor mass was significantly smaller in the *B. longum* 420 group than in the *B. longum* 2012 group on days 17, 19, and 21 after leukemia cell inoculation (*P* < 0.01, *P* < 0.001, and *P* < 0.001, respectively; Fig. 1a); hence, the antitumor activity of *B. longum* 420 was shown to be caused by WT1-specific immune responses. Furthermore, tumor size on day 17 after leukemia cell inoculation was significantly smaller in the *B. longum* 105-A and 2012 groups than in the PBS group (*P* < 0.05 each; Fig. 1a).

### In vivo CD8^+^ T cell-dependent antitumor activity of ***B. longum*** 420

On day 25 after leukemia cell inoculation, tumor size was significantly smaller in the *B. longum* 420 group than in the PBS group (*P* < 0.05). However, tumor size became nearly equal between the anti-CD8 antibody + *B. longum* 420 group and the PBS group (Fig. 1b). Namely, the administration of anti-CD8 antibody counterbalanced the antitumor activity of the *B. longum* 420 group, indicating that CD8^+^ T cells are the main effector of the *B. longum* 420 group.

### Infiltrations of CD4^+^ and CD8^+^ T cells in the C1498-mWT1 mass

The infiltrations of CD4^+^ and CD8^+^ T cells in the C1498-mWT1 mass (CD4^+^ or CD8^+^ tumor-infiltrating lymphocytes [TILs]) are shown in Fig. 1c, and the numbers of CD4^+^ and CD8^+^ TILs in Fig. 1d and Fig. 1e, respectively. The number of CD4^+^ T cells was significantly greater in the *B. longum* 420 group than in the PBS and *B. longum* 105-A groups (*P* < 0.01 and *P* < 0.05; Fig. 1d). The number of CD8^+^ T cells was significantly greater in the *B. longum* 420 group than in the PBS, *B. longum* 105-A, and *B. longum* 2012 groups (*P* < 0.001, *P* < 0.01, and *P* < 0.01; Fig. 1e). Namely, mice treated with *B. longum* 420 showed the most intense intratumor infiltration of CD4^+^- and CD8^+^- T cells.

### Frequency of WT1-specific CTLs and production of anti-WT1 protein IgG antibody

In a tetramer assay that detects WT1-specific CTLs which recognize WT1_126-134_ peptide (i.e., the H-2D^b^-restricted epitope of WT1 protein), we then determined WT1-specific CTL frequency in CD8^+^ T cells collected from PBMCs. The frequency of WT1-specific CTLs significantly greater in the *B. longum* 420 group than in the PBS, *B. longum* 105-A, and *B. longum* 2012 groups (*P* < 0.01 each; Fig. 1f); the representative data thereof are shown in Fig. 1g. The cytotoxic activity (as expressed in % specific lysis) of splenocytes on WT1_126-134_-pulsed RMAS cells was shown in the *B. longum* 420 group but not in the other study 3 groups (Fig. 1h).

CD4^+^ T cell help is considered to activate and/or expand CTLs. We assessed the activation of WT1-specific CD4^+^ T cells by examining the production of the anti-WT1 IgG antibody because CD4^+^ T cells stimulate the class-switch of IgM to IgG [19]. At week 4, absorbance at a wavelength of 550 nm reflecting the titers was significantly greater in the *B. longum* 420 group than in other study groups (*P* < 0.05 each; Fig. 1i).

### FISH assay of *B. longum* in the PPs

**Fig. 2:**
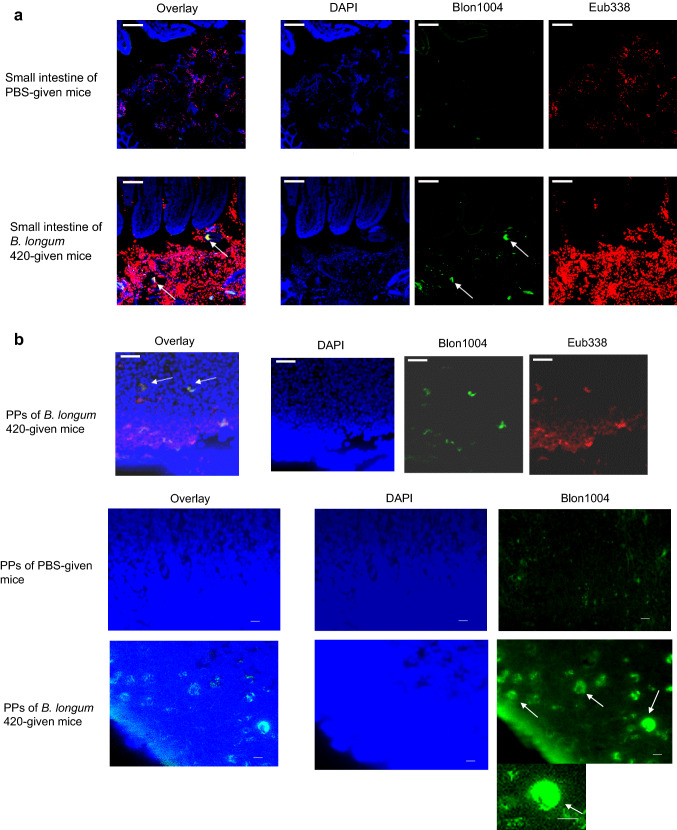
Localization of bacterial rRNA in the small intestine including Peyer’s patches. Mice were treated orally with PBS and *B. longum* 420 on days 1 to 10. **a** On day 11, animals were killed to remove the small intestine. FISH of the rRNA of *B. longum* 420 taken in the small intestinal lumen and walls of mice treated with PBS or *B. longum* 420 by using DAPI (blue), as well as probes—Blon1004 (green) and Eub338 (red). Representative data in triplicate are shown. Scale bar, 10 µm. Arrows, the rRNA of *B. longum* 420 taken in the small intestinal lumen. **b** On day 11, animals were killed to conduct FISH of the rRNA of *B. longum* 420 taken in by DCs in the PPs by using DAPI (blue), as well as probes—Blon1004 (green) and Eub338 (red). Representative data in triplicate are shown. Scale bar, 10 µm. Arrows, bacterial rRNA taken in by DCs. Magnification (bar: 20 µm) to indicate the better presentation of cytoplasmic fluorescence rRNA, ribosomal ribonucleic acid; FISH, fluorescence in situ hybridization; DAPI, 4′6-diamidino-2-phenylindol; PBS, phosphate-buffered saline; PPs, Peyer’s patches; DCs, dendritic cells

4′6-Diamidino-2-phenylindole (DAPI) and probes—Blon1004 (Cy3-labeled) and Eub338 (Cy5-labeled)—were used to conduct the multicolor FISH of the small intestine from mice in an attempt to determine the uptake thereof into the intestine including the PPs as manifested in blue, green, and red, respectively (Fig. 2). *B. longum* 420 was found not only in the lumen of the small intestine (Fig. 2a) but also in DCs from the PPs (Fig. 2b) in the FISH images.

**Fig. 3:**
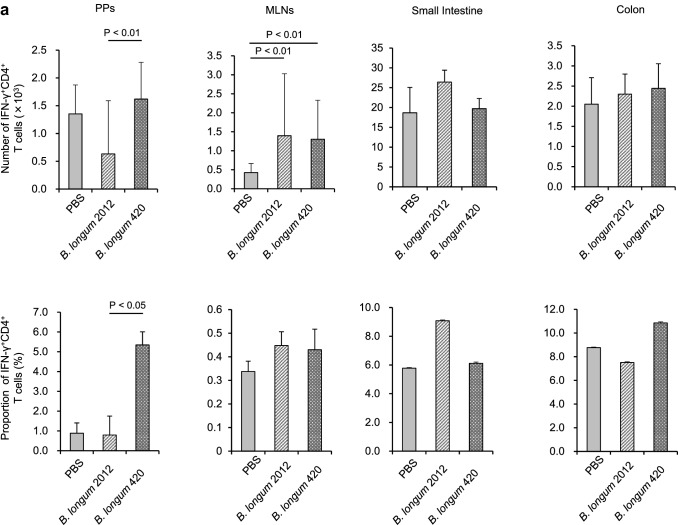

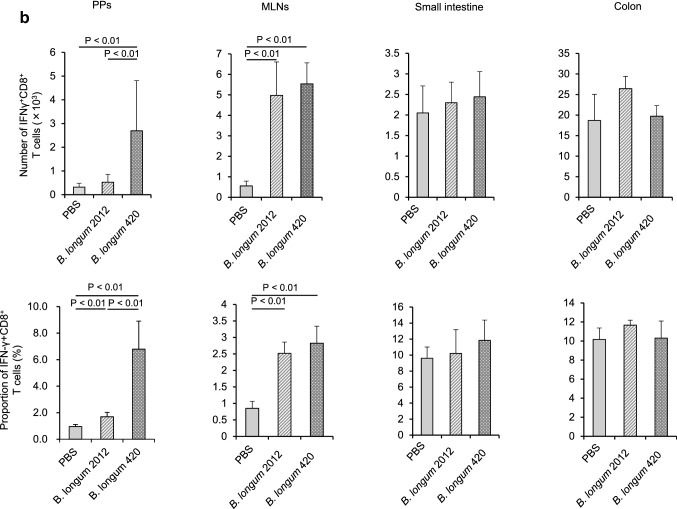
Immunization with *Bifidobacterium*. **a** Oral administration of study materials was conducted on days 1 to 28. On day 29, the number and proportion of IFN-γ^+^CD4^+^ T cells in the PPs, MLNs, small intestine, and colon were determined (*n* = 8/group). Scale bars represent SE. **b** The number and proportion of IFN-γ^+^CD8^+^ T cells in the PPs, MLNs, small intestine, and colon were determined (*n* = 8/group). Scale bars represent SE IFN, interferon; PBS, phosphate-buffered saline; PPs, Peyer’s patches; MLNs, mesenteric lymph nodes

Next, we examined the mechanisms of intestinal immunity generating the antitumor activity of *B. longum*. Tumor-nonbearing mice were orally given PBS, *B. longum* 2012, or *B. longum* 420 for 4 weeks. On day 29, cells were collected from the PPs, MLNs, small intestine, and colon, followed by the stimulation thereof with PMA and calcium ionophore to examine the production of IFN-γ (Fig. 3a and b, respectively). The numbers and proportions of IFN-γ^+^CD4^+^ T cells in the PPs were significantly greater in the *B. longum* 420 group than in the *B. longum* 2012 group (*P* < 0.05 or < 0.01; Fig. 3a, b), which indicated the greater functionality of CD4^+^ T cells in the PPs for mice in the *B. longum* 420 group than for mice in the *B. longum* 2012 group.

The numbers and proportions of IFN-γ^+^CD4^+^ and IFN-γ^+^CD8^+^ T cells in the MLNs were significantly greater in the *B. longum* 2012 and 420 groups than in the PBS group, excepting the proportion of IFN-γ^+^CD8^+^ T cells (*P* < 0.05 or < 0.01; Fig. 3b), which indicated the greater antitumor functionality of CD4^+^ and CD8^+^ T cells in the MLNs for mice in the *B. longum* 2012 and 420 groups than for mice in the PBS group.

### Activation of DCs by intestinal immunity

**Fig. 4:**
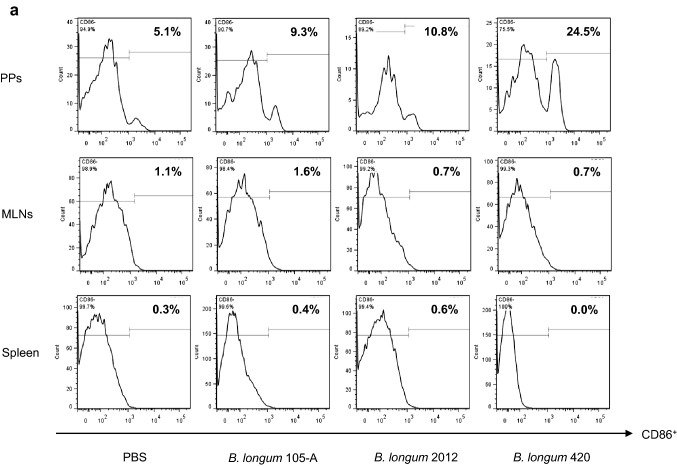

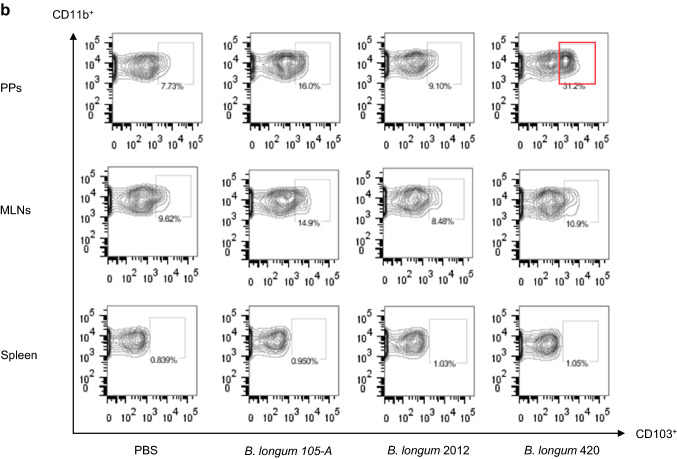

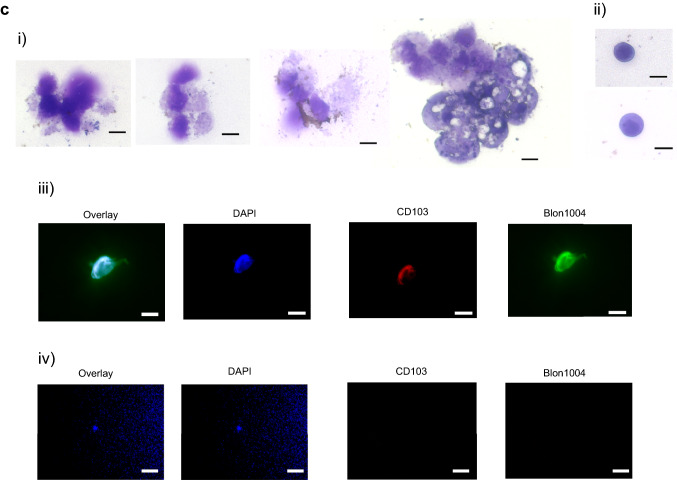
Staining of dendric cells in the Peyer’s patches, MLN and spleen. Representative data in triplicate are shown. Oral administration of *B. longum* 420 was conducted on days 1 to 10, and mice were killed on day 11. **a** Flow cytometry indicating the proportion of CD86^+^ DCs in the PPs, MLNs, and spleen. **b** Flow cytometry indicating the proportion of CD11b^+^CD11c^+^CD103^+^ DCs in the PPs, MLNs, and spleen. On day 15 of oral administration, two aliquots of DCs were collected from the PPs of mice that were treated with *B. longum* 420. One aliquot of CD11c^+^ DCs sorted from the PPs was used to conduct May-Giemsa staining: 4 representative, activated DCs **c**-i) and 2 inactivated DCs **c**-ii). Another aliquot of representative, activated DCs **c**-iii) and inactive DCs **c**-iv)—which were stained with the anti-CD103-APC antibody (M290, BD Biosciences)—were deposited onto a slide according to the cytospin method before their hybridization to conduct FISH using DAPI (blue), CD103 (red), and Blon1004 (green). Representative data in triplicate are shown. Scale bar, 2 µm. DCs, dendritic cells; PP, Peyer’s patches; FISH, fluorescence in situ hybridization; DAPI, 4′6-diamidino-2-phenylindol

The proportion of CD86^+^ DCs among MHC class II^+^CD11c^+^ DCs in the PPs was greater for *B. longum* 105-A (9.3%) and 2012 (10.8%) than for PBS (5.1%) and was markedly greater for *B. longum* 420 (24.5%) than for other 2 bacterial strains (Fig. 4a). Furthermore, the proportion of CD103^+^CD11b^+^CD11c^+^ DCs was greater for *B. longum* 105-A (16.0%) and 2012 (9.10%) than for PBS (7.73%) (Fig. 4b) and was markedly greater for *B. longum* 420 (31.2%) than for other 2 bacterial strains. These CD103^+^ DCs, which captured bacterial rRNA, were found more abundant in the PPs from mice in the *B. longum* 420 group, implying the good recognition and presentation of a TAA—WT1—by DCs in the entry of the lymphocytic network leading to systemic circulation. Activated DCs in the PPs, which were stained by May-Giemsa staining, are shown in Fig. 4c (i), and inactive ones in Fig. 4c (ii). The presence of Blon1004-labeled *B. longum* 420 taken in by CD103^+^CD11c^+^ DCs in the PPs (Fig. 4c (iii)) corroborates the flowcytometric data of Fig. 4b in contrast to CD103^−^CD11c^+^ DCs that did not take in *B. longum* 420. Figure 4c (iv) indicates the nonuptake of *B. longum* 420 by CD103^−^ DCs resulting in a failure to activate DCs.

## Discussion

The present study elucidated immunological mechanisms by which this *B. longum*-based oral anticancer vaccine triggers intestinal immunity in mice, identified immunocompetent cells in the immune system—PPs and MLNs, and afforded the following findings: (1) CD8^+^ T cells played the major role in the cytotoxic activity of *B. longum* 420, with the significantly greater activity compared to *B. longum* 2012; (2) only *B. longum* 420 induced WT1-specific CTLs; (3) CD4^+^ T cell help was evidenced by the production of anti-WT1 IgG antibody and by the increased number and proportion of CD4^+^ T cells capable of producing IFN-γ; (4) *B. longum* 420 taken in by DCs in the PPs induced the generation of CD103^+^CD11b^+^CD11c^+^ DCs capable of efficiently presenting WT1 peptides on the cell surface to prime CD8^+^ T cells; and (5) the activation of DCs by *B. longum* strains and by the add-on effect of WT1 anchoring.

CD8^+^ T cells played the major role in the antitumor activity of *B. longum* 420 because the administration of the anti-CD8 antibody completely suppressed the activity to mice in the *B. longum* 420 group. CD8^+^ T cells significantly infiltrated in the tumor mass of mice in the *B. longum* 420 group compared to those in the *B. longum* 105-A and 2012 groups. The frequency of WT1-specific CTLs in PBMCs is significantly greater in the former group than in the latter groups. The antitumor activity of *B. longum* 420 is WT1-specific because the cytotoxic activity of CD8^+^ T cells on WT1 peptide-pulsed RMAS cells increased in the *B. longum* 420 group, implying that CD8^+^ T cells collected from mice in the *B. longum* 420 group recognize WT1 peptide and are capable of lysing WT1-expressing tumor cells. Namely, CD8^+^ T cells are the principal immunologic competent cells that exerted the antitumor activity of *B. longum* 420. Furthermore, the production of anti-WT1 IgG antibody in the *B. longum* 420 group infers the involvement of CD4^+^ T cell help.

*B. longum* DNA contains immunostimulatory motifs that trigger an innate immunity [20], and three *B. longum* strains examined in the present study activated DCs in the PPs and suggested the antigen-independent antitumor activity thereof based on innate immunity.

Of special note was the finding that *B. longum* 420 exerted antitumor activity despite the fact that the proportion of CD103^+^ DCs—which have conventionally been considered to reduce intestinal immune responses by stimulating regulatory T cells in the intestine [21]—was increased by *B. longum* 420. Our data suggest that WT1 peptides cross-presented by CD103^+^ DCs in the PPs to CD8^+^ T cells prime or reactive CTLs in the systemic circulation via the MLNs, leading to the exertion of the antitumor activity of *B. longum* 420, as inferred by a previous study [22].

Besides WT1-specific T cell immunity, we demonstrated that T cell immunity in the PPs and MLNs was enhanced by two *B. longum*-based oral vaccines. Subsequent to the stimulation of cells from the PPs and MLNs by PMA and calcium ionophore, the number and proportion of IFN-γ^+^CD4^+^ T cells in the PPs from mice were significantly greater in the *B. longum* 420 group than in the *B. longum* 2012 group, which indicated that CD4^+^ T cells in the PPs from mice were functionally more active in the *B. longum* 420 group than in the *B. longum* 2012 group. This finding is consistent with our demonstration that more DCs are activated in the PPs from mice in the *B. longum* 420 group than in the *B. longum* 2012 group, since activated DCs stimulated both WT1-specific and -nonspecific CD4^+^ T cells in the PPs. Furthermore, CD4^+^ T cells in the MLNs from mice were functionally more active in the *B. longum* 2012 and 420 groups than in the PBS group; however, the reason for this finding was unknown because the proportions of CD103^+^ DCs in the MLNs were equivalent between the *B. longum* 2012 and 420 groups. Oral vaccination with *B. longum* strains may stimulate T cells in the MLNs due to unelucidated mechanisms.

In association with the above-mentioned activation states of T cells in the PPs and MLNs, the *B. longum* 420 group showed the most intense intratumoral infiltration of CD4^+^ and CD8^+^ T cells presumably to due to (1) the nonspecific activation of DCs by *Bifidobacterium* itself and to (2) the priming of WT1-specific, cytokine-producing CD4^+^ and CD8^+^ T cells that are activated by DCs in the PPs which, in turn, further stimulates these DCs. The efficient activation of DCs and immunological priming of CD8^+^ T cells by *B. longum* 420 at the PPs and MLNs appear to have contributed to the exertion of the antitumor activity thereof. Further investigation is needed to confirm this speculation.

In contrast to a *Lactococcus lactis* oral vaccine expressing human papillomavirus type 16 E7 under development for exogenous viruses [23], our oral vaccine has the potential of clinical applicability to many solid and hematologic malignancies of endogenous origin. However, further research is required to investigate differences in the phenotypic features of CD103^+^ DCs with antitumor activity in the intestinal and systemic immune systems.

In conclusion, the present study indicates that this oral anticancer vaccine triggers intestinal immunity to exert the antitumor activity of WT1-specific CD8^+^ T cells possibly through CD4^+^ T cell help, and we speculate that unidentified components of the systemic immune system (e.g., the function of natural killer cells and macrophages, as well as phenotypes of CD4^+^ T cells) appear to be involved in the antitumor activity of the vaccine. This oral WT1 protein vaccine has clinical applicability because of its potential advantages: high safety, low-cost manufacturing, easy scale-up for massive production, and expectation for good adherence by cancer patients, especially pediatric patients.

## Data Availability

All data are available in the manuscript on a reasonable request. Correspondence should be addressed to Yoshiko Hashii, MD, PhD (yhashii@ped.med.osaka-u.ac.jp).
